# Tongue volume in adults with skeletal Class III dentofacial deformities

**DOI:** 10.1186/s13005-016-0110-4

**Published:** 2016-03-22

**Authors:** N. Ihan Hren, U. Barbič

**Affiliations:** Department of Maxillofacial and Oral Surgery, University Medical Centre Ljubljana, Zaloška cesta 2, 1000 Ljubljana, Slovenia

**Keywords:** Skeletal Class III, Tongue, Volume

## Abstract

**Background:**

The size of the tongue is implicated as an essential etiological factor in the development of malocclusions. The aim of our study was to assess tongue size in skeletal Class III (SCIII) patients in comparison to adults with normal occlusion, using three-dimensional (3D) ultrasound.

**Methods:**

The SCIII group consisted of 54 subjects; 34 females and 20 males and the control group contained 36 subjects, 18 from each gender with Class I relationship. 3D ultrasound images of the tongues were acquired, and then the tongues’ volumes were assessed.

**Results:**

The males in both the SCIII and control groups had significantly larger tongue volumes than the female subjects (mean SCIII 100.8 ± 6.3 and control 92.4 ± 9.8 cm^3^ in males vs. SCIII 77.4 ± 10.2 and control 67.2 ± 5.6 cm^3^ in females). The highly significantly larger tongue volumes were in SCIII patients of both genders (p were less than 0.01 for female and 0.03 for male). The tongue volumes within the whole SCIII group were significantly larger with more negative Wits values.

**Conclusion:**

The tongue volumes are significantly bigger in SCIII subjects than normal. Larger tongues correlate with more severe SCIII. The clinical importance of this data is that limited mandibular setback planning is necessary to prevent narrowing of respiratory airways.

## Background

Skeletal class III (SCIII) better known as mandibular prognathism in spite the fact that this term describes only one form of this dentofacial deformity. It is a severe dentofacial disharmony which frequently shows combinations of skeletal and dentoalveolar characteristics resulting in different facial appearances. The common characteristic is changed anteroposterior relationship between maxilla and mandible with their changed sizes and positions in relation to the anterior cranial base. The more common clinical sign of that is Class III dental malocclusion.

The etiology of SCIII appears to be a result of interactions amongst the genetically determined factors and many internal and external environmental factors but the precise roles of both have as yet to be clarified. Amongst them traditionally the muscle equilibrium among intraoral and buccal forces has been understood for the normal development of dental arches but this simple theory was revisited because any equilibrium between the force of the tongue and the force of lips couldn’t be found [1].

It is hypothesised that the tongue volume, besides posture and function, is of crucial importance in the etiology of malocclusions and dentofacial deformities [2]. Macroglossia and its consequences are well-known in Beckwith-Wiedemann syndrome [3] and acromegaly [4], it was suggested as a possible cause of open bite and mandibular prognathism [5]. However the role of tongue volume in mandibular prognathism was also rejected [6]. The studies of tongue reductions have reported about changing the skeletal Class III into Class I in the early preadolescent period [7] and about dental arch lingual collapse after a decrease in tongue volume [8]. Clinical studies have reported that tongue volume is correlated with mandibular arch size [9], vertical facial height, and chin position [6].

The dilemma is longstanding about tongue adaptation to existing oral morphology or actively moulding its surrounding tissues [10, 11]. So determining the size of the tongue in different facial morphological variants and the examination of other possible tongue roles such as pressure, mobility, and rest postures are still to be clarified. It has also been suggested that an increase in the volume of soft tissues induces osteogenic reaction at the growth site of the bone. With the relative increase of tongue volume, which means a decreased volume of the oral cavity as consequence of orthognathic surgical procedures with normal volume of the tongue, the relapse can be explained and indications for tongue reductions determined [12].

The aim of this study was to evaluate the tongue size at its normative volume in SCIII patients in comparison to patients with normal occlusion using three-dimensional ultrasound as valid and non-invasive methods. Therefore, the null hypothesis assumes that there is insignificant correlation between the tongue volume and the maxilla-mandibular relationship CIII.

## Methods

The study was approved by the Ethics Committee of the Republic of Slovenia, and informed consents were obtained from all subjects involved in the study. The SCIII group consisted of 54 subjects, 34 females (aged 25.5 ± 10.3 years) and 20 males (aged 21.5 ± 4.6 years), with Class III molar relationships, negative overjet and skeletal deformity CIII regarding the lateral cephalometric analysis (Wits values less than −1). The control group consisted of 36 subjects with no reborn or acquired facial deformities, 18 females (aged 24.7 ± 2.2 years) and 18 males (aged 26.0 ± 3.7 years), with Class I molar relationships and normal anterior teeth overjet and overbite. Both groups were matched with comparable body mass and height. All the subjects were Slovenian, meaning of Caucasian ancestry.

On the basis of height (cm) and weight (kg) at the time of the 3D ultrasound tongue image acquisitions their bodies’ mass indexes (BMI) were calculated using the formula BMI = weight (kg)/height (m^2^) for all participants in the study. The basic data for all the studied subjects are presented in Table 1.

**Table 1:** The participants in the study; females and males individually in the SCIII group and controls, their number, average values and standard deviations according to age, SCIII characteristics (SNA, SNB, Wits), and their body mass indexes (BMI)

Group	Sex	Age (mean ± SD)	BMI (mean ± SD)	SNA (mean ± SD)	SNB (mean ± SD)	WITS (mean ± SD)
	Female	25.5 ± 10.3	21.9 ± 2.7	80.9 ± 4.6	83.5 ± 4.7	−8.5 ± 3.5
RIII	Male	21.5 ± 4.6	26.3 ± 4.6	82.7 ± 4.4	88.6 ± 5.1	−13.3 ± 4.6
	Total	24.0 ± 8.8	23.6 ± 4.1	81.6 ± 4.5	85.4 ± 5.4	−10.3 ± 4.6
	Female	24.7 ± 2.2	20.6 ± 1.6			
Control	Male	26.0 ± 3.7	24.5 ± 2.4			
	Total	25.3 ± 3.1	22.5 ± 2.8			

**Fig. 1:**
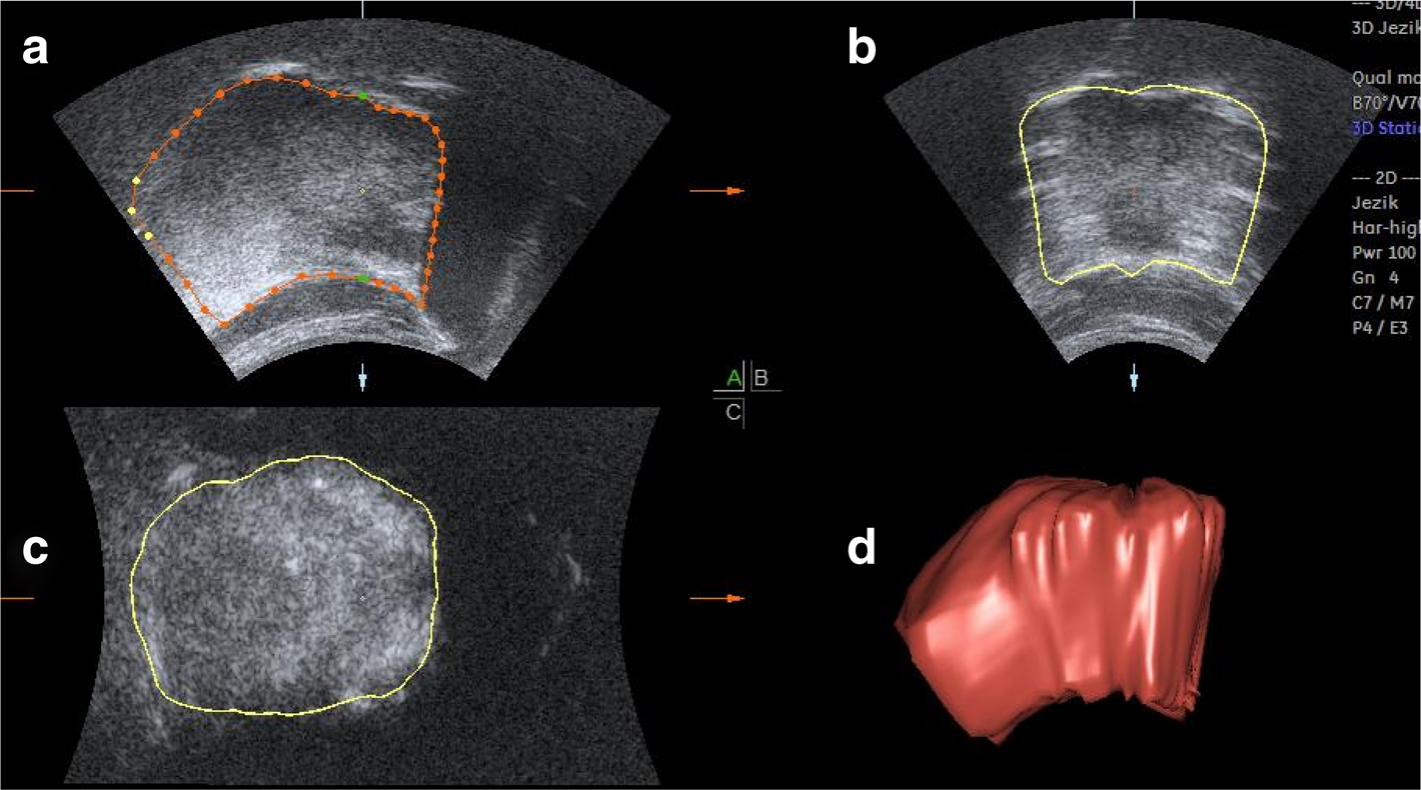
Ultrasound image of the tongue; multi-planar view with boundaries (**a** sagittal cross-section, **b** transversal cross-section in dorsum area, **c** transversal cross-section in apical area, **d** three-dimensional US reconstruction)

The ultrasound investigation were carried out on relaxed subjects sitting in upright positions with their heads fixed using the Frankfurt horizontal line parallel to the ground floor. The Voluson 730 expert ultrasonographic device (Kretztechnik, Zipf, Austria) and a 3D convex transducer (RAB 2–5.Mhz) were used. The transducer was positioned on the midsagittal line on the mouth’s floor skin submentally. A 3D/4D mode setting (3D static render, max. quality, vol-angle70°) for data acquisition was selected. Each tongue was recorded twice (Fig. 1).

The tongue volume assessment was performed using 4D VIEW program software (GE Healthcare, Kretztechnick, Zipf, Austria). By using VOCAL II (Virtual Organ Computer-aided Analysis) application, the tongues’ borders were determined manually on tongue images; on twelve sections of the same tongue obtained by turning the tongue over 180° around its vertical axis through the centre. Each step was taken by rotation of the image plane for 15°. The outline of the tongue followed the curvilinear surface of the dorsum of the tongue, including the genioglossus muscle and following the boundary between the tongue and the floor of the mouth. Then the volumes of the outlined tongues were calculated, the final estimations were done as the average volumes of 2 measured volumes.

For statistical analysis, SPSS for Windows version 18 (SPSS Inc., Chicago IL) was used for statistical analysis. The Student *t*-test was performed in order to evaluate the gender differences regarding the tongues’ volumes and comparisons between the SCIII groups and the controls. Using linear multivariate regression (Table 3) we tested the influences of the observed parameters on the tongues’ volumes in the SCIII group. The results were considered to be significant at a 5% level (*p* < 0.05).

## Results

**Fig. 2:**
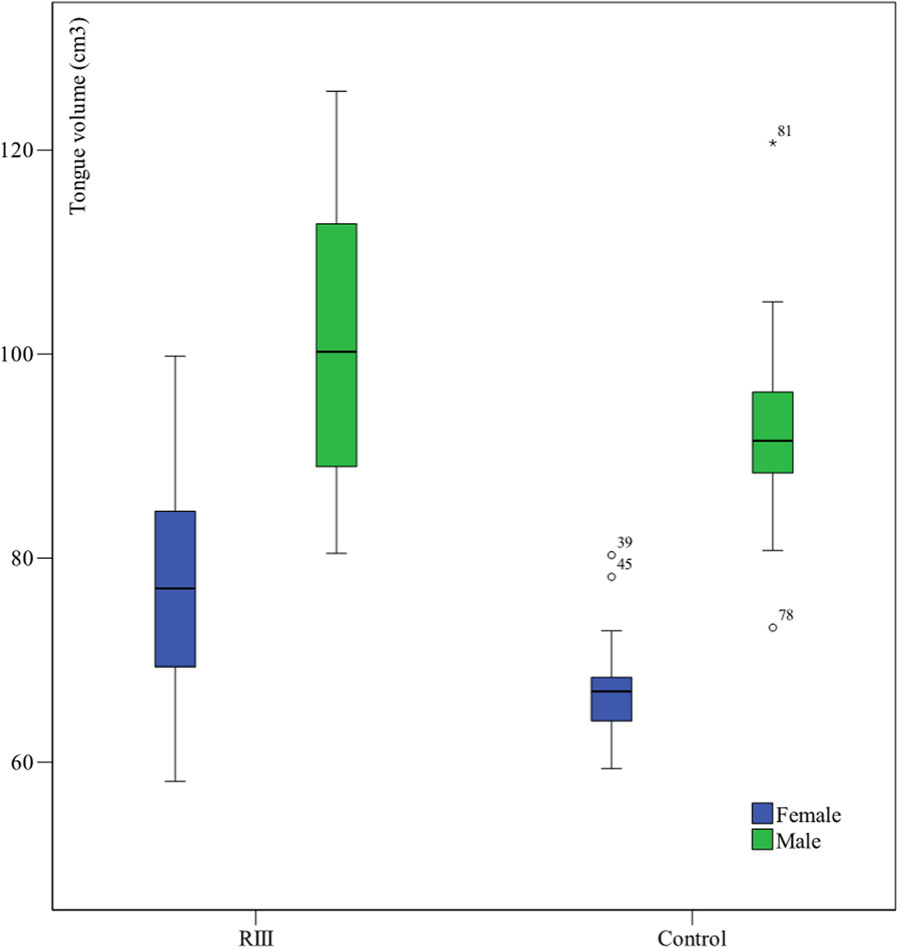
The tongues’ volumes in cm^3^ for the observed groups

The tongues volumes (mean values and standard deviations - SD) are presented in Table 2. In view of evident sexual dimorphism, we decided on gender specific comparisons among the SCIII and control groups. These data are graphically presented in Fig. 2.

**Table 2:** The tongues’ volumes in the SCIII and control groups (total, female and male) in cm^3^ (average values - mean, standard deviations- SD) and the same values for BMI characteristics for each group

Group	Sex	Tongue volume (mean ± SD)	BMI (mean ± SD)
	Female	77.4 ± 10.2	21.9 ± 2.7
RIII	Male	100.8 ± 12.9	26.3 ± 4.6
	Total	86.1 ± 15.9	23.6 ± 4.1
	Female	67.2 ± 5.6	20.6 ± 1.6
Control	Male	92.4 ± 9.8	24.5 ± 2.4
	Total	79.8 ± 15.0	22.5 ± 2.8

The comparisons (Independent Samples *T*-Test) among the SCIII and controls are presented in Table 3.

**Table 3:** The statistical comparisons between the equal groups with SCIII and controls with *t*-test for Equality of Means

Sex	t	Sig.	Mean Difference	Std. Error Difference
All	1.89	0.06	6.25	3.31
Female	3.93	0.00	10.21	2.60
Male	2.26	0.03	8.36	3.69

Using linear multivariate regression (Table 4) we tested the influences of the observed parameters on tongues’ volumes in the SCIII group. The only significance was the influence of Wits and BMI values when observing the whole group. BMI had a positive influence (the higher the BMI, the bigger the tongues), on the one hand, and the Witt values had a negative influence (the more negative values, the bigger the tongues) on the other hand.

**Table 4:** Linear multivariate analysis with tongue volume as the dependent parameter SNA, SNB, WITS, BMI and age were checked for their impacts

Female				Male			All		
beta		t	Sig.	beta	t	Sig.	beta	t	Sig
(Constant)	64.45	1.90	0.07	26.069	0.35	0.74	17.87	0.50	0.62
SNA	1.09	1.32	0.20	0.754	0.81	0.43	0.82	1.22	0.23
SNB	−1.29	−1.59	0.12	> −0.01	> −0.01	0.99	−0.64	−0.93	0.36
WITS	−1.45	−1.95	0.06	0.16	0.13	0.90	−1.57	−2.30	**0.03**
BMI	0.75	1.10	0.28	0.74	1.04	0.32	1.78	3.80	**0.00**
Age	0.14	0.83	0.41	−0.25	−0.33	0.75	−0.14	−0.71	0.48

Bold-numbers show statistically significant values (p ≤ 0.05)

**Fig. 3:**
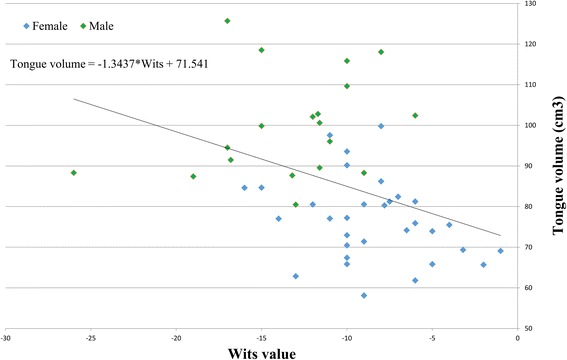
Relationship between the tongues’ volumes (cm3) and the Wits values for the SCIII subjects is presented. The trend of bigger tongue volumes in subjects with smaller (more negative) Wits values is obvious. The fit line for the whole group with its features (correlation coefficient −1.34 and constant) is presented too

The connection between the Wits values and the tongues’ volumes alone for the SCIII subjects was checked and is graphically presented in Fig. 3. The Fit line is drawn and the correlation coefficient calculated (−1.34).

## Discussion

A large tongue was regarded as a possible cause of mandibular prognathism by Köle in 1965 [13], and on many further occasions. They also found that those subjects with mandibular prognathism showed tongue volumes similar to those of the control group [6]. So the exact role of the tongue in SCIII patients had not been clarified as yet. However, the majority of this data were without accurate quantitative information on tongues’ volumes.

As the tongue is encased within the oral cavity when at rest, direct measurement of its real dimensions within the oral cavity is difficult. Therefore, various techniques have been developed for evaluating the tongue’s size in vivo: direct tongue measurements [14], different impression techniques [9] and the fluid displacement method [15] but were unable to measure the interior’ portion of the tongue. Furthermore, different imaging techniques have been introduced to assess the tongue volume: cephalometrics [16], computed tomography (CT) [17], cone-beam computed tomography (CB-CT) [18] and magnetic resonance imaging (MRI) [19]. However, these techniques have different disadvantages.

Two-dimensional (2D) ultrasound being a non-invasive, radiation-free procedure, is used for the assessment of tongue function as well as for the evaluation of tongue thickness [20] and the estimation of tongue volume [21], while three-dimensional (3D) ultrasound is already used for the assessment of tongue posture [22], as a tool for the evaluation of tongue function [23]. The examination is rather quick and can be repeated due to its non-radiation characteristic. The ultrasound provides a clear image of the tongue’s surface and makes it possible to distinguish the tongue’s musculature from the mouth’s floor musculature [21, 24]. The image is not obscured by teeth because the transducer is placed submentally and the beam directed upward bypasses the teeth. Moreover, the advantage of 3D ultrasound is the possibility of digitally storing an entire tissue volume sample. Using 4D View with VOCAL II application the tongue’s borders can be manually determined and the volume of the reconstructed tongue calculated. In our previous study we demonstrated that 3D ultrasound assessment of the tongue’s volume is highly reliable in terms of intra-class correlation coefficients of repeatability, as well as of intra-rated and inter-rated reliability.

In our study the tongue volumes expressed significant gender dimorphism as in the majority of similar studies with the exception of the study by Uysal T et al. [18]. The subjects in the male groups of SCIII and the controls had significantly larger tongue volumes than the subjects in the female groups. Generally, the results of the assessed tongues’ volumes of the control group in our study are comparable to the findings of other studies. Liégeois et al. [25] presented similar tongue volumes for male and female groups by MRI, also the results estimated from the sagittal MRI images in Lauder et al. [26] research are comparable but they hadn’t been focused on occlusion. Furthermore, Iida-Kondo et al. [19] calculated similar tongue volumes with MRI in male adults with normal occlusion only. Nevertheless, these results differ from the results of the Do et al. [27] study, in which the tongues’ volumes in a group of patients without sleep-disordered breathing were much larger. The differences may be explained due to different anatomical outlines, as the extrinsic tongue muscles, mylohyoid and anterior bellies of the digastric muscle were included within the study. In contrast, less than half of our values for tongue volumes were observed by Uysal T. [18] but the reason was that on CB-CT images they artificially determined the posterior border of the tongue and only measured the anterior part of the mobile tongue.

The comparisons between objective tongue volumes in the SCIII patients could not be done properly because of the lack of data. Yoo E et al. [6] didn’t find any differences among the tongue volumes in Japanese females with and without mandibular prognathism, but the number of subjects was relatively small and they didn’t correlate the results using Wits or ANB values for further determining this relationship. Tamari K et al. [9] reported that tongue volumes and lower dental arch sizes were significantly correlated with what in some way described similar findings to ours but his results were found on Japanese adults with normal occlusion. The highly significant larger tongue volumes in the SCIII patients in our study were a surprise for us because of our opposite expectations at the beginning of this study.

We haven’t correlated the tongue volumes with hyoid bone positions, which should be clarified in the future. The reason for that is that the constant relation tongue – the hyoid bone is already known from obstructive sleep apnea studies [28]. The more anterior positions of the hyoid bone in SCIII patients were described by Yamaoka M et al. [29] and this fact they connected with describing the influence of the tongue as pseudo-macroglossia. They didn’t measure any tongue size parameter and the results for CIII were done by comparing with CII patients and not subjects with normal occlusion. In addition the stability of mandibular setback was connected by the stability of the hyoid position [30] which is connected by the tongue. The upper and forward displacements of the hyoid bone immediately after mandibular setback and later repositioning [31] is known too. There is a study that the adaptation of the hyoid bone position and tongue mass to the altered environment after mandibular setback surgery may preclude the necessity for downsizing the tongue mass against relapses in patients with normal tongue morphologies [12]. However, the results of our study that significantly increased tongue volumes are presented in more severe SCIII patients, demand different thoughts.

The tongue volumes we cannot simply connect with tongue pressure because we don’t know much about adaptive tongue capacities and its tonus changes. The interesting data were that 12 months after the tongue size reductions, the pressures of the tongue on the teeth didn’t differ significantly from the pre-surgical values but the resting pressures were lower than before surgery and were closer to those of the reference sample [10]. A well- known study recommended that in the cases of increased tongue-tip pressure after mandibular setback, tongue reduction for stability is necessary [32]. They observed that in the majority of cases this pressure decreases after surgery and then returns to approximately its original level. The fact is also that the tongue pressure may depend on many factors such as the breathing mode and body position, all these are connected by the position of the hyoid bone [33].

In our study, the tongues’ volumes significantly correlated with BMI and with the Wits values for the whole observed SCIII group. The correlation with BMI was the expected one. The significant correlation with Wits value was quite a surprise but more severe SCIII dentofacial deformities with more negative Wits values (bigger absolute numbers) were obviously connected with larger tongues. By taking into consideration the large groups used during this valid and accurate method, we think that tongue volumes could deduce SCIII or represent important etiological factors. For orthognathic surgery larger tongue volumes represent an additional powerful reason why large setbacks of the mandibles with passive backward positioning of the tongue could be an important cause of sleep apnea. Sleep apnea had not been primarily described as an consequence of these procedures [34], but later the long-term narrowing of airway space after bilateral sagittal split osteotomy has been noticed several times [35, 36]. Surgical reduction of large tongue volumes is often avoided because of potential operative or postoperative problems; correction of SCIII patients with more negative Wits values and larger tongues is then left to correction with osteotomies. In such cases, the simultaneous use of maxillary advancement and a lesser mandibular set-back offers less detrimental influence on the airway space and less tendency toward skeletal relapse than a more extensive mandibular set-back alone.

## Conclusions

The subjects in the male groups of SCIII and controls had significantly larger tongue volumes than the female subjects in both groups. The highly significantly larger tongue volumes were in both genders in the SCIII patients. Even more, the tongue volumes in the SCIII group were significantly larger in more negative Wits values – which means that larger tongue volumes are significant for more severe SCIII. The clinical importance of this is that careful mandibular setback planning is necessary.

## References

[1] Proffit WR (1978). Equilibrium theory revisited: factors influencing position of the teeth. Angle Orthod.

[2] Brodie AG (1953). Muscular factors in the diagnosis and treatment of malocclusions*. Angle Orthod.

[3] Clauser L, Tieghi R, Polito J (2006). Treatment of macroglossia in Beckwith-Wiedemann syndrome. J Craniofac Surg.

[4] Gosau M, Vogel C, Moralis A, Proff P, Kleinheinz J, Driemel O (2009). Mandibular prognathism caused by acromegaly - a surgical orthodontic case. Head Face Med.

[5] Xin N, Tao W, Ashwin DV, Jinlin S (2015). Establishment of integral biomechanical balance in the correction of tongue source skeletal dentomaxillofacial open bite deformities. J Craniofac Surg.

[6] Yoo E, Murakami S, Takada K, Fuchihata H, Sakuda M (1996). Tongue volume in human female adults with mandibular prognathism. J Dent Res.

[7] Miyawaki S, Oya S, Noguchi H, Takano-Yamamoto T (2000). Long-term changes in dentoskeletal pattern in a case with Beckwith-Wiedemann syndrome following tongue reduction and orthodontic treatment. Angle Orthod.

[8] Liu ZJ, Shcherbatyy V, Gu G, Perkins JA (2008). Effects of tongue volume reduction on craniofacial growth: A longitudinal study on orofacial skeletons and dental arches. Arch Oral Biol.

[9] Tamari K, Shimizu K, Ichinose M, Nakata S, Takahama Y (1991). Relationship between tongue volume and lower dental arch sizes. Am J Orthod Dentofacial Orthop.

[10] Frohlich K, Ingervall B, Schmoker R (1993). Influence of surgical tongue reduction on pressure from the tongue on the teeth. Angle Orthod.

[11] Ingervall B, Schmoker R (1990). Effect of surgical reduction of the tongue on oral stereognosis, oral motor ability, and the rest position of the tongue and mandible. Am J Orthod Dentofacial Orthop.

[12] Kawakami M, Yamamoto K, Noshi T, Miyawaki S, Kirita T (2004). Effect of surgical reduction of the tongue on dentofacial structure following mandibular setback. J Oral Maxillofac Surg.

[13] Köle H. Results, experience, and problems in the operative treatment of anomalies with reverse overbite (mandibular protrusion). Oral Surg Oral Med Oral Pathol. 1965;19(4):427–50. http://dx.doi.org/10.1016/003\0-4220(65)90002-2.

[14] Oliver RG, Evans SP (1986). Tongue size, oral cavity size and speech. Angle Orthod.

[15] Bandy HE, Hunter WS (1969). Tongue volume and the mandibular dentition. Am J Orthod.

[16] Cuccia AM, Campisi G, Cannavale R, Colella G (2007). Obesity and craniofacial variables in subjects with obstructive sleep apnea syndrome: comparisons of cephalometric values. Head Face Med.

[17] Roehm EG (1982). Computed tomographic measurement of tongue volume relative to its surrounding space. Am J Orthod.

[18] Uysal T, Yagci A, Ucar FI, Veli I, Ozer T (2013). Cone-beam computed tomography evaluation of relationship between tongue volume and lower incisor irregularity. Eur J Orthod.

[19] Iida-Kondo C, Yoshino N, Kurabayashi T, Mataki S, Hasegawa M, Kurosaki N (2006). Comparison of tongue volume/oral cavity volume ratio between obstructive sleep apnea syndrome patients and normal adults using magnetic resonance imaging. J Med Dent Sci.

[20] Capilouto GJ, Frederick ED, Challa H (2012). Measurement of infant tongue thickness using ultrasound: a technical note. J Clin Ultrasound.

[21] Wojtczak JA (2012). Submandibular sonography: assessment of hyomental distances and ratio, tongue size, and floor of the mouth musculature using portable sonography. J Ultrasound Med.

[22] Volk J, Kadivec M, Music MM, Ovsenik M (2010). Three-dimensional ultrasound diagnostics of tongue posture in children with unilateral posterior crossbite. Am J Orthod Dentofacial Orthop.

[23] Bressmann T, Thind P, Uy C, Bollig C, Gilbert RW, Irish JC (2005). Quantitative three-dimensional ultrasound analysis of tongue protrusion, grooving and symmetry: data from 12 normal speakers and a partial glossectomee. Clin Linguist Phon.

[24] Shawker TH, Sonies BC, Stone M (1984). Soft tissue anatomy of the tongue and floor of the mouth: an ultrasound demonstration. Brain Lang.

[25] Liegeois F, Albert A, Limme M (2010). Comparison between tongue volume from magnetic resonance images and tongue area from profile cephalograms. Eur J Orthod.

[26] Lauder R, Muhl ZF (1991). Estimation of tongue volume from magnetic resonance imaging. Angle Orthod.

[27] Do KL, Ferreyra H, Healy JF, Davidson TM (2000). Does tongue size differ between patients with and without sleep-disordered breathing?. Laryngoscope.

[28] Battagel JM, Johal A, L’Estrange PR, Croft CB, Kotecha B (1999). Changes in airway and hyoid position in response to mandibular protrusion in subjects with obstructive sleep apnoea (OSA). Eur J Orthod.

[29] Yamaoka M, Furusawa K, Uematsu T, Okafuji N, Kayamoto D, Kurihara S (2003). Relationship of the hyoid bone and posterior surface of the tongue in prognathism and micrognathia. J Oral Rehabil.

[30] Wickwire NA, White RP, Proffit WR (1972). The effect of mandibular osteotomy on tongue position. J Oral Surg.

[31] Jorge TM, Brasolotto AG, Goncales ES, Filho HN, Berretin-Felix G (2009). Influence of orthognathic surgery on voice fundamental frequency. J Craniofac Surg.

[32] Wickwire NA, Proffit WR (1972). Changes in tongue position and activity following mandibular osteotomy. Am J Orthod.

[33] Takahashi S, Ono T, Ishiwata Y, Kuroda T (1999). Effect of changes in the breathing mode and body position on tongue pressure with respiratory-related oscillations. Am J Orthod Dentofacial Orthop.

[34] Riley RW, Powell N, Guilleminault C (1987). Current surgical concepts for treating obstructive sleep apnea syndrome. J Oral Maxillofac Surg.

[35] Abdelrahman TE, Takahashi K, Tamura K, Nakao K, Hassanein KM, Alsuity A (2011). Impact of different surgery modalities to correct class III jaw deformities on the pharyngeal airway space. J Craniofac Surg.

[36] Greco JM, Frohberg U, Van Sickels JE (1990). Long-term airway space changes after mandibular setback using bilateral sagittal split osteotomy. Int J Oral Maxillofac Surg.

